# Associations between leukocyte telomere length and three measures of folate status: a cross-sectional analysis of NHANES 1999–2002

**DOI:** 10.3389/fnut.2025.1714482

**Published:** 2026-01-09

**Authors:** Qian Xue, Hongju Chen

**Affiliations:** 1 Department of Hepatobiliary Surgery, People's Hospital of Leshan, Leshan, Sichuan, China; 2 Department of Gynecology, Leshan Hospital of Traditional Chinese Medicine, Leshan, Sichuan, China

**Keywords:** leukocyte telomere length, folate, NHANES, cross-sectional study, dietary folate intake

## Abstract

**Background:**

Shortening of leukocyte telomere length (LTL) is a core hallmark of cellular senescence. Folate provides essential methyl groups for DNA synthesis and repair, theoretically capable of slowing telomere attrition by maintaining genomic integrity. However, current epidemiological studies on folate and LTL are still limited, and most prior investigations have relied on a single metric of folate status.

**Objective:**

This study aimed to investigate the associations between LTL and three measures of folate status, dietary intake, serum folate and red blood cell (RBC) folate, to provide more precise epidemiological evidence of folate's role in cellular aging and to establish a scientific basis for potential nutritional intervention strategies.

**Methods:**

This cross-sectional analysis included 7,324 participants from the National Health and Nutrition Examination Survey (NHANES) 1999–2002. Multivariable linear regression models, adjusted for demographic, lifestyle, and nutritional factors, were used to assess associations. Restricted cubic splines and piecewise linear regression were employed to evaluate non-linear relationships.

**Results:**

After full adjustment, both serum and RBC folate showed positive linear associations with longer LTL (*P* for trend < 0.05). Dietary folate exhibited a non-linear relationship with LTL (*P* for non-linearity < 0.05). Meeting the recommended intake (≥400 μg/day) was associated with longer telomeres (β = 0.05, 95% CI: 0.03–0.06). A saturation effect was observed; beyond 500.86 μg/day (95% CI: 490.71–511.01), further intake did not significantly increase LTL.

**Conclusion:**

Adequate folate status is associated with longer telomeres, supporting a role in mitigating cellular aging. Dietary intake ≥400 μg/day is beneficial, but exceeding ~500 μg/day offers no further advantage.

## 1 Introduction

Leukocyte telomere length (LTL) is a well-established biomarker of cellular senescence (1). Its shortening correlates with the number of cell divisions and ultimately contributes to cellular aging (2, 3). Shorter LTL is closely associated with an increased risk of age-related diseases such as cardiovascular disease, diabetes, and cancer (4). Therefore, identifying modifiable factors that slow telomere shortening is of significant importance for promoting healthy aging (5).

As a central cofactor in one-carbon metabolism, folate supplies methyl groups essential for DNA synthesis and repair (6). Theoretically, it could mitigate telomere shortening by maintaining telomeric DNA integrity or modulating telomerase activity (7, 8). However, impaired folate metabolism may lead to homocysteine accumulation, which in turn could accelerate telomere attrition through oxidative stress and inflammatory pathways (9). Therefore, there is an urgent need for large-scale epidemiological evidence to clarify the relationship between folate and LTL.

To address these controversies, this study utilizes a multidimensional approach to assess folate status: dietary intake reflects long-term habits, serum folate indicates recent exposure, and red blood cell (RBC) folate represents long-term bodily storage (10). By simultaneously examining the associations of these three indicators with LTL, we aim to provide more comprehensive and robust scientific evidence regarding the role of folate in cellular aging, thereby contributing to the theoretical basis for nutrition-focused strategies to promote healthy aging.

## 2 Materials and methods

### 2.1 Study population

**Figure 1:**
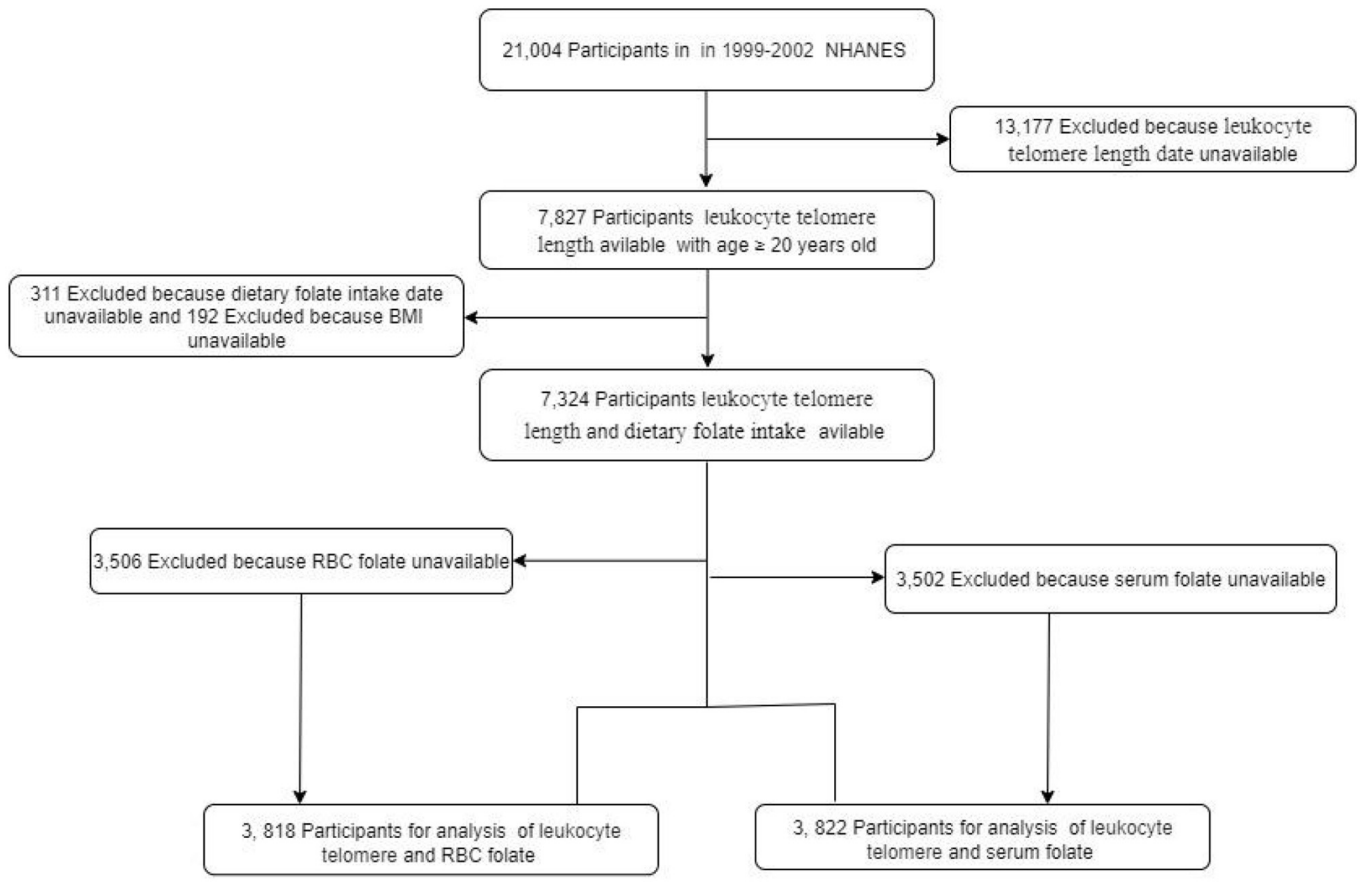
Flow chart of the study.

The NHANES is a cross-sectional study designed to assess the health and nutritional status of the non-institutionalized U.S. civilian population. The initial analytical cohort for this study consisted of all participants aged 20 years or older from the combined 1999–2002 cycles who had valid measurements of LTL (*n* = 7,827). Given that the three primary exposures (dietary, serum, and RBC folate) had different patterns of missing data, we constructed three analytical samples to maximize the use of available data and the sample size for each analysis: For the analysis of dietary folate, we excluded participants with missing data on dietary folate intake (*n* = 311) or body mass index (BMI, *n* = 192), resulting in a final sample of 7,324 participants. For the analysis of serum folate, from the initial cohort, we excluded those missing serum folate data (*n* = 3,502), resulting in a final sample of 3,822 participants. For the analysis of RBC folate, from the initial cohort, we excluded those missing RBC folate data (*n* = 3,506), resulting in a final sample of 3,818 participants. A flowchart illustrating the participant selection process is provided in Figure 1. All NHANES protocols were reviewed and approved by the Ethics Review Board of the National Center for Health Statistics, and all participants provided written informed consent.

### 2.2 Leukocyte telomere length (LTL)

In the NHANES study, the methods for measuring LTL have been detailedly reported (11, 12). Briefly, whole blood samples from respondents aged 20 and above were included for DNA analysis: DNA was extracted from whole blood at the National Center for Health Statistics (NCHS) laboratory and shipped to a contracted laboratory for telomere length assessment. LTL was measured by quantitative polymerase chain reaction (qPCR), expressed as the T/S ratio relative to a standard reference DNA (i.e., relative LTL). The testing protocol involved three replicate measurements per sample (each replicate included two technical measurements, totaling six measurements) to calculate the mean and standard deviation of the T/S ratio for each participant. For quality control, each sample was initially tested at least twice; if the coefficient of variation (CV) of the T/S ratio between the two tests was ≤ 7%, the average was taken as the result; if the CV exceeded 7%, a third test was conducted, and the average of the two closest values was used as the final result. It should be noted that the T/S ratio is proportional to the average LTL, and for ease of understanding, it is referred to as “LTL” in this study.

### 2.3 Dietary folate intake, serum folate, and RBC folate

Dietary folate intake, referring to the daily dietary intake of total folate, was accurately calculated from the NHANES 24-h food recall questionnaire, which detailed the types and amounts of food consumed by the subjects (13, 14), as well as from the dietary interview procedure manual (15, 16). Participants were categorized into quartiles (Q1–Q4) based on their dietary folate intake. According to the World Health Organization's recommended daily intake of 400 μg of folate, the participants were divided into two groups: the folate deficiency group (< 400 μg), and the folate adequate group (≥400 μg).

Blood samples were processed, stored, and transported to the Division of Laboratory Sciences, Environmental Health Laboratory, National Center for Environmental Health, Centers for Disease Control and Prevention for analysis. Serum folate was measured using liquid chromatography-tandem mass spectrometry (LC-MS/MS), while RBC folate was determined by microbiological assay.

### 2.4 Covariates

To ensure the accuracy of the research analysis, this study included as many relevant variables as possible. The variables analyzed were age, race/ethnicity, education level, family poverty income ratio (PIR), marital status, smoking status, physical activity, BMI, and energy intake.

Race/ethnicity was categorized as Non-Hispanic White, Non-Hispanic Black, Mexican American, or other race. Marital status was categorized as married/with a partner or alone. Education level was categorized based on the number of years of education received: less than 9 years, 9–12 years, or more than 12 years. Physical activity was classified as sedentary, moderate, or vigorous. Smoking status was categorized as never smokers, current smokers, and former smokers. BMI was classified as normal, overweight or obese with 25 and 30 kg/m^2^, and energy was calculated based on the NHANES 24-h food recall questionnaire and dietary interview procedure manual.

### 2.5 Statistical analyses

This study utilized data from the 1999–2002 cycles of the NHANES. All analyses incorporated appropriate survey weights (specifically, the mobile examination center [MEC] weights) to account for the complex sampling design and ensure national representativeness. The recommended four-year MEC weight (WTMEC4YR) was applied to the combined 1999–2002 dataset. Continuous variables are presented as the mean ± standard deviation if normally distributed, or as the median (interquartile range) if skewed. Categorical variables are summarized as frequencies and percentages (%). Folate measures—dietary intake, serum concentration, and RBC concentration—were analyzed both as continuous variables and as categorical variables based on quartiles. The associations between these folate measures and LTL were assessed using weighted multivariable linear regression, and results are expressed as beta coefficients (β) with their 95% confidence intervals (CIs). The linearity of these associations was examined using restricted cubic splines. Missing data for covariates—including educational attainment (*n* = 10), marital status (*n* = 364), family PIR (*n* = 661), smoking status (*n* = 13), and physical activity (*n* = 6)—were handled using multiple imputation. This process generated ten imputed datasets. A sensitivity analysis using complete-case analysis was conducted to verify the robustness of the primary findings.

Model 1 was the crude model. Model 2 was adjusted for age and gender. Building on Model 2, Model 3 was further adjusted for demographic characteristics, including educational attainment, marital status, race/ethnicity, and family PIR. Model 4 extended Model 3 by additionally adjusting for BMI, smoking status, physical activity, and energy intake.

The statistical software program used for all analyses was R 3.3.2 (http://www.R-project.org, The R Foundation, Shanghai, China). Additionally, Free Statistics Software 1.9 (Beijing Free Clinical Medical Technology Co., LTD) was employed for data processing and analysis. A descriptive analysis was conducted for all participants. Results with *p* < 0.05 were considered statistically significant.

## 3 Results

### 3.1 Baseline characteristics of the study population

Table 1 presents the baseline characteristics of the 7,324 study participants, who were categorized into quartiles based on dietary folate intake. The overall mean age of the participants was 49.0 years. A higher proportion of males was observed in the high folate intake group, whereas the low intake group contained a higher proportion of females. Additionally, participants in the high folate intake group tended to be younger and had higher daily energy intake. This group also included a greater proportion of individuals with higher educational attainment, those who were married or cohabiting, those with higher family PIR, and those who engaged in higher levels of physical activity.

**Table 1:** Baseline characteristics of study participants, stratified by dietary folate intake.

**Characteristic**	**Participants**					
	**Total (*****n** =* **7,324)**	**Q1 (*****n** =* **1,825) (0–228** μ**g)**	**Q2 (*****n** =* **1,823) (229–340** μ**g)**	**Q3 (*****n** =* **1,839) (341–489** μ**g)**	**Q4 (*****n** =* **1,837) (**>**489** μ**g)**	***p-value***
**Gender**						< 0.001
Male	3,520 (48.1)	678 (37.2)	791 (43.4)	904 (49.2)	1,147 (62.4)	
Female	3,804 (51.9)	1,147 (62.8)	1,032 (56.6)	935 (50.8)	690 (37.6)	
Age (years)	49.0 ± 18.5	51.1 ± 18.7	49.4 ± 18.6	49.3 ± 18.7	46.3 ± 17.7	< 0.001
**Race/ethnicity**						< 0.001
Non-Hispanic White	3,715 (50.7)	791 (43.3)	951 (52.2)	991 (53.9)	982 (53.5)	
Non-Hispanic Black	1,229 (16.8)	452 (24.8)	311 (17.1)	240 (13.1)	226 (12.3)	
Mexican American	1,772 (24.2)	438 (24)	410 (22.5)	458 (24.9)	466 (25.4)	
Others	608 (8.3)	144 (7.9)	151 (8.3)	150 (8.2)	163 (8.9)	
**Education level (years)**						< 0.001
< 9	1,133 (15.5)	354 (19.4)	276 (15.1)	257 (14)	246 (13.4)	
9–12	3,005 (41.0)	843 (46.2)	767 (42.1)	723 (39.3)	672 (36.6)	
>12	3,186 (43.5)	628 (34.4)	780 (42.8)	859 (46.7)	919 (50)	
**Marital status**						< 0.001
Married or living with a partner	4,729 (64.6)	1,069 (58.6)	1,195 (65.6)	1,233 (67)	1,232 (67.1)	
Living alone	2,595 (35.4)	756 (41.4)	628 (34.4)	606 (33)	605 (32.9)	
**Family PIR**						< 0.001
Low	2,031 (27.7)	644 (35.3)	491 (26.9)	444 (24.1)	452 (24.6)	
Medium	2,848 (38.9)	742 (40.7)	708 (38.8)	721 (39.2)	677 (36.9)	
High	2,445 (33.4)	439 (24.1)	624 (34.2)	674 (36.7)	708 (38.5)	
**Physical activity**						< 0.001
Sedentary	3,256 (44.5)	968 (53)	813 (44.6)	778 (42.3)	697 (37.9)	
Moderate	1,911 (26.1)	433 (23.7)	471 (25.8)	509 (27.7)	498 (27.1)	
Vigorous	2,157 (29.5)	424 (23.2)	539 (29.6)	552 (30)	642 (34.9)	
**Smoking status**						< 0.001
Never	3,778 (51.6)	917 (50.2)	921 (50.5)	986 (53.6)	954 (51.9)	
Former	1,967 (26.9)	426 (23.3)	509 (27.9)	504 (27.4)	528 (28.7)	
Current	1,579 (21.6)	482 (26.4)	393 (21.6)	349 (19)	355 (19.3)	
**BMI (kg/m** ^2^ **)**						< 0.001
Normal	2,302 (31.4)	527 (28.9)	565 (31)	574 (31.2)	636 (34.6)	
Overweight	2,670 (36.5)	603 (33)	662 (36.3)	702 (38.2)	703 (38.3)	
Obese	2,352 (32.1)	695 (38.1)	596 (32.7)	563 (30.6)	498 (27.1)	
Energy (kcal/day)	2,119.9 ± 1,025.9	1,367.1 ± 587.7	1,899.5 ± 680.8	2,261.6 ± 778.1	2,944.6 ± 1,215.3	< 0.001
LTL	1.0 ± 0.3	1.0 ± 0.3	1.0 ± 0.3	1.0 ± 0.3	1.0 ± 0.3	< 0.001

BMI, body mass index; LTL, leukocyte telomere length; PIR, poverty income ratio; Q1–Q4, quartiles, based on dietary folate intake.

Data are presented as mean (SD) or *n* (%).

### 3.2 Association between folate and LTL

To investigate the association between folate and LTL, we constructed four multivariate linear regression models. When dietary folate intake was analyzed as a continuous variable, a positive association with LTL was observed in Models 1–3 (*P* < 0.05) (Table 2).

**Table 2:** Association between dietary folate intake and LTL.

**Dietary folate (ug)**	**No. of total**	**Model 1**		**Model 2**		**Model 3**		**Model 4**	
		β**-95%CI**	***P*** **-value**	β**-95%CI**	***P*** **-value**	β**-95%CI**	***P*** **-value**	β**-95%CI**	***P*** **-value**
**Overall**									
Dietary folate (100 ug)	7,324	0.01 (0–0.01)	0.001	0 (0–0.01)	0.04	0 (0–0.01)	0.018	0 (0–0.01)	0.062
**Quartile**									
Q1	1,825	0(Ref)		0(Ref)		0(Ref)		0(Ref)	
Q2	1,823	0.01 (−0.01–0.03)	0.403	0 (−0.02–0.02)	0.854	0 (−0.02–0.03)	0.651	0.01 (−0.02–0.03)	0.653
Q3	1,839	0.02 (−0.01–0.04)	0.13	0.01 (−0.01–0.04)	0.251	0.02 (−0.01–0.04)	0.139	0.02 (−0.01–0.04)	0.143
Q4	1,837	0.05 (0.03–0.08)	< 0.001	0.03 (0.01–0.06)	0.003	0.04 (0.02–0.06)	0.001	0.04 (0.01–0.07)	0.003
*P* for trend			< 0.001		0.002		0.001		0.002
**Dietary folate**									
< 400 ug	4,519	0(Ref)		0(Ref)		0(Ref)		0(Ref)	
≥400 ug	2,805	0.05 (0.03–0.06)	< 0.001	0.04 (0.02–0.05)	< 0.001	0.04 (0.02–0.06)	< 0.001	0.05 (0.03–0.06)	< 0.001

Model 1 was not adjusted. Model 2 was adjusted for sociodemographic variables (age and gender). Model 3 was adjusted for Model 2+race, education level, marital status, family PIR, Model 4 was adjusted for Model 3+smoking status, physical activity, BMI and energy consumption.

LTL, leukocyte telomere length; CI, confidence interval; Q, quartile of dietary folate intake, Q1 (0–228 μg), Q2 (229–340 μg), Q3 (341–489 μg), and Q4 (>489 μg).

When dietary folate intake was categorized into quartiles (Q1: 0–228 μg; Q2: 229–340 μg; Q3: 341–489 μg; Q4: >489 μg), a significant positive trend was observed across all models (P for trend < 0.05). In the Model 4, the β coefficients were 0.01 (95% CI: −0.02–0.03) for Q2, 0.02 (95% CI: −0.01–0.04) for Q3, and 0.04 (95% CI: 0.01–0.07) for Q4, when compared to Q1. When participants were categorized based on the international recommended daily intake of 400 μg, those consuming ≥400 μg had significantly longer telomeres in all models. In Model 4, the difference was β = 0.05 (95% CI: 0.03–0.06; *P* < 0.001) (Table 2). These results were robust in a sensitivity analysis using complete-case analysis (see Supplementary Table S1). In subgroup analyses by gender, age, smoking status, and BMI, the positive association between dietary folate intake ≥400 μg/day and longer LTL was consistent, with no statistically significant effect modification observed (all *P* for interaction >0.05; see Supplementary Table S2).

A positive correlation was also observed between serum folate (as a continuous variable) and LTL in all models (*P* < 0.05). When analyzed by quartiles (Q1: 1.0–9.2 nmol/L; Q2: 9.3–13.3 nmol/L; Q3: 13.4–18.0 nmol/L; Q4: >18.0 nmol/L), higher serum folate levels were associated with increased LTL. In Model 4, compared to Q1, the β values were 0.03 (95% CI: 0.01–0.05) for Q2, 0.04 (95% CI: 0.02–0.07) for Q3, and 0.03 (95% CI: 0.01–0.05) for Q4 (Table 3).

**Table 3:** Association between serum folate, RBC folate and LTL.

**Blood folate (nmol/L)**	**No. of total**	**Model 1**		**Model 2**		**Model 3**		**Model 4**	
		β**-95%CI**	***P*** **-value**	β**-95%CI**	***P*** **-value**	β**-95%CI**	***P*** **-value**	β**-95%CI**	***P*** **-value**
Overall	3,822								
Serum folate		0 (0–0)	0.001	0 (0–0)	0.04	0 (0–0)	0.018	0 (0–0)	0.062
**Quartile**									
Q1	937	0(Ref)		0(Ref)		0(Ref)		0(Ref)	
Q2	973	0.01 (−0.01–0.03)	0.414	0.03 (0–0.05)	0.019	0.03 (0.01–0.05)	0.009	0.03 (0.01–0.05)	0.012
Q3	955	0.02 (−0.01–0.04)	0.218	0.05 (0.02–0.07)	< 0.001	0.05 (0.03–0.07)	< 0.001	0.04 (0.02–0.07)	< 0.001
Q4	957	−0.04 (−0.06 to −0.01)	0.002	0.04 (0.01–0.06)	0.002	0.04 (0.02–0.06)	0.001	0.03 (0.01–0.05)	0.01
*P* for trend			0.004		0.001		< 0.001		0.005
Overall	3,818								
RBC folate		0 (0–0)	< 0.001	0 (0–0)	0.032	0 (0–0)	0.012	0 (0–0)	0.017
**Quartile**									
Q1	952	0(Ref)		0(Ref)		0(Ref)		0(Ref)	
Q2	953	0 (−0.02 to 0.03)	0.915	0.01 (−0.01 to 0.03)	0.28	0.02 (0–0.04)	0.123	0.02 (−0.01 to 0.04)	0.128
Q3	961	−0.01 (−0.04 to 0.01)	0.333	0.03 (0.01–0.05)	0.014	0.03 (0.01–0.06)	0.003	0.03 (0.01–0.06)	0.004
Q4	952	−0.05 (−0.07 to −0.02)	< 0.001	0.03 (0.01–0.05)	0.015	0.04 (0.01–0.06)	0.003	0.03 (0.01–0.06)	0.004
*P* for trend			< 0.001		0.006		0.001		0.002

Model 1 was not adjusted. Model 2 was adjusted for sociodemographic variables (age, gender). Model 3 was adjusted for Model 2+race, education level, marital status, family PIR, Model 4 was adjusted for Model 3+smoking status, physical activity, BMI and energy consumption.

LTL, leukocyte telomere length; CI, confidence interval; RBC, red blood cell; Q, quartile of serum folate, Q1 (1.0–9.2 nmol/L), Q2 (9.3–13.3 nmol/L), Q3 (13.4–18.0 nmol/L), and Q4 (>18.0 nmol/L); Q, quartile of RBC folate; Q1(28–219 nmol/L), Q2(220–282 nmol/L), Q3(283–367 nmol/L), Q4(>367 nmol/L).

Similarly, when analyzed as a continuous variable, RBC folate was positively associated with LTL in all models (*P* < 0.05). When categorized into quartiles (Q1: 28–219 nmol/L; Q2: 220–282 nmol/L; Q3: 283–367 nmol/L; Q4: >367 nmol/L), a significant positive trend was observed (*P* for trend < 0.05). In the Model 4, compared to Q1, the β values were 0.02 (95% CI: −0.01–0.04) for Q2, 0.03 (95% CI: 0.01–0.06) for Q3, and 0.03 (95% CI: 0.01–0.06) for Q4 (Table 3).

These results indicate that higher folate status, measured through dietary intake, serum concentration, or RBC concentration, is consistently associated with longer LTL.

### 3.3 Non-linear and linear associations of folate measures with LTL

**Figure 2:**
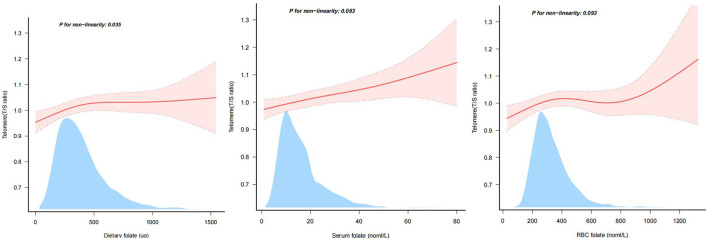
Linear relationship between folate and LTL. LTL, leukocyte telomere length; Adjusted Model was adjusted for age, gender, race/ethnicity, education level, marital status and family PIR, smoking status, physical activity, BMI and energy consumption. Only 99.8% of the date is displayed.

As summarized in Table 2, dietary folate was positively associated with LTL. To further characterize this relationship, we performed restricted cubic spline analyses. After excluding extreme outliers (the top 0.2% of dietary folate intake), the analysis showed a significant non-linear relationship between dietary folate and LTL in the fully adjusted model (*P* for non-linearity = 0.035). In contrast, both serum and RBC folate showed linear positive associations with LTL (*P* for non-linearity >0.05) (Figure 2).

### 3.4 Saturation point of dietary folate at ~500 μg/day

**Figure 3:**
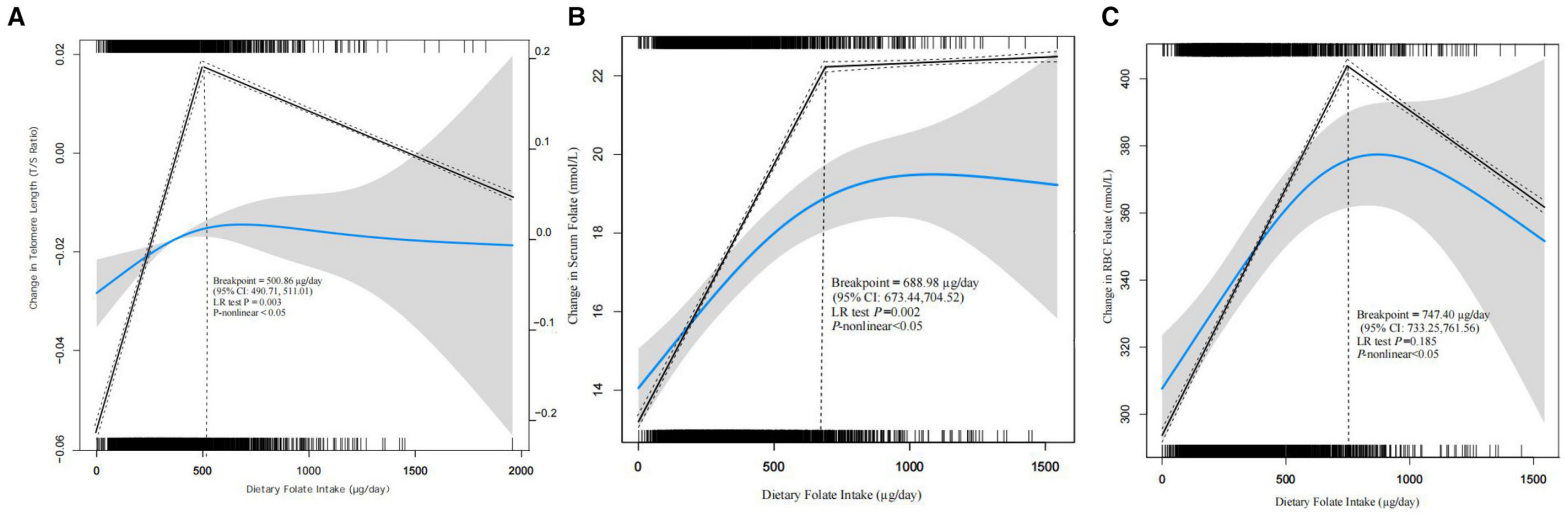
**(A)** Breakpoint analysis of dietary folate and LTL. **(B)** Breakpoint analysis of serum and dietary folate. **(C)** Breakpoint analysis of RBC and dietary folate. LTL, leukocyte telomere length; CI, confidence interval; LR, likelihood ratio. Adjusted Model was adjusted for sociodemographic variables (age and gender), race/ethnicity, education level, marital status and family PIR, smoking status, physical activity, BMI and energy consumption. Only 99.8% of the date is displayed.

Piecewise linear regression analysis, performed after excluding extreme outliers (top 0.2% of dietary folate intake), revealed a significant non-linear relationship between dietary folate and LTL (*P* for non-linearity < 0.05). This model, which included a break-point, fitted the data significantly better than a simple linear model (likelihood ratio test *P* = 0.003). The inflection point was identified at 500.86 μg/day (95% CI: 490.71–511.01). This indicates that LTL increases with folate intake up to approximately 500 μg/day, beyond which it plateaus and further intake provides no significant additional benefit (Figure 3A).

### 3.5 Correlation between dietary folate and biomarkers

To validate the dietary folate assessment and explore the dose-response relationship, we examined the associations of dietary folate intake with serum and RBC folate concentrations. Piecewise linear regression revealed significant non-linear relationships for both biomarkers (*P* for non-linearity < 0.05). For serum folate, a strong linear association was observed up to an inflection point of 688.98 μg/day (95% CI: 673.44–704.52). Beyond this point, the rate of increase attenuated and was no longer statistically significant. While a break-point was detected for RBC folate at 747.40 μg/day, the association remained positive and significant across all intakes, and the non-linear model was not a statistically better fit (LR test *P* = 0.185) (Figures 3B, C).

## 4 Discussion

Previous epidemiological studies examining the association between folate and LTL have predominantly relied on single indicators such as serum folate, RBC folate, or dietary intake (17–19). This methodological approach makes it challenging to comprehensively characterize the complex panorama of folate metabolism. In order to address this limitation, the present study represents the first investigation within a nationally representative large cohort to simultaneously examine the association between LTL and three core indicators: dietary folate intake, serum folate, and RBC folate. This multidimensional research design facilitates the validation of previous fragmented findings and reveals the unique patterns of action among different folate indicators, thereby providing a more comprehensive assessment.

Our distinct findings for different folate metrics are well-supported by their underlying biology. The stable linear associations observed for both serum and RBC folate with longer LTL align with the constitutive role of folate in one-carbon metabolism. As an essential cofactor, folate provides precursors for the synthesis of thymidine and purines (20, 21), which are the fundamental building blocks for DNA replication and repair. Telomeres, as specialized DNA structures, are critically dependent on the fidelity of these processes for their maintenance (22, 23). Therefore, adequate systemic folate levels may delay telomere shortening by ensuring a sufficient supply of substrates for telomeric DNA synthesis (24, 25). The absence of a “ceiling effect” for these biomarkers suggests a continuous, dose-dependent relationship. This view is corroborated by experimental and epidemiological evidence. Li et al. demonstrated that folic acid supplementation inhibits telomere depletion in mice (26), while Fan et al. reported that higher maternal folate levels are associated with longer telomeres in newborns (27). Furthermore, folate deficiency leads to elevated homocysteine (28), a metabolite known to induce oxidative stress and inflammation—both established accelerants of telomere shortening. Thus, the linear protective effect of serum and RBC folate likely operates through dual pathways: supporting continuous DNA synthesis and mitigating oxidative damage (29).

The most significant public health finding is the discovery of a pronounced non-linear relationship between dietary folate intake and LTL, exhibiting clear diminishing returns. Beyond approximately 500.86 μg/day, the upward trend in LTL response to increased intake markedly flattens. Mechanistic analysis indicates that the conversion of dietary folate to serum folate reaches saturation at approximately 688.98 μg/day, while the conversion to erythrocyte folate exhibits an inflection point or even reversal at around 747.40 μg/day. This finding indicates the presence of inherent physiological upper limits with regard to human folate absorption, conversion, and storage. The potential aetiological factors may include the saturation of relevant enzyme systems (e.g., dihydrofolate reductase) (30, 31)or imbalanced interactions with other nutrients (e.g., vitamin B12) (32, 33). Consequently, as dietary intake increases persistently, the marginal benefit in elevating active folate levels and telomere protection diminishes significantly. Our findings have important implications for public health guidelines. The current Recommended Dietary Allowance (RDA) for adults is typically 400 μg/day, while the Tolerable Upper Intake Level (UL) is 1,000 μg/day (34, 35). Our study identifies an optimal intake level of approximately 500 μg/day for telomere health, which lies between these two benchmarks. This suggests that while achieving the RDA is crucial, exceeding 500 μg/day through high-dose supplementation may offer little additional benefit for cellular aging and could represent an unnecessary metabolic load. Future dietary guidelines could consider providing a more refined upper range within the safe limit to optimize health outcomes.

Limitations warrant attention: The cross-sectional design precludes establishing causality. Measurements of serum and RBC folate were available only for a subpopulation that was not fully representative, posing a risk of selection bias. Although biological mechanisms support folate's influence on telomeres, reverse causation (e.g., poorer nutritional status among those with shorter LTL) or residual confounding (e.g., genetic polymorphisms, oxidative stress, and inflammation) cannot be entirely ruled out.

## 5 Conclusion

This study indicates that adequate folate status is independently associated with longer leukocyte telomere length among US adults, supporting the notion that it may potentially delay cellular aging. In view of the saturation effect that has been observed, it is recommended that future dietary guidelines, whilst continuing to emphasize the RDA, consider incorporating approximately 500 μg/day as an optimal intake reference to achieve health benefits whilst avoiding metabolic saturation. It is recommended that this hypothesis be tested further through the execution of prospective studies.

## Data Availability

The original contributions presented in the study are included in the article/Supplementary material, further inquiries can be directed to the corresponding author.
