# Supplementary material for: Associations between leukocyte telomere length and three measures of folate status: a cross-sectional analysis of NHANES 1999–2002
Source: Front Nutr. 2026 Jan 9;12:1714482. doi: 10.3389/fnut.2025.1714482 (PMC12827169; doi:10.3389/fnut.2025.1714482)
Supplement: Supplementary file 1 [file Table_1.docx]

**Supplementary Table S1  Sensitivity Analysis: Association between dietary folate intake and LTL using complete-case analysis (n = 6,343).**

| **Dietary folate （ug）** | **No.of**  **total** | **Model 1** | | **Model 2** | | **Model 3** | | **Model 4** | |
| --- | --- | --- | --- | --- | --- | --- | --- | --- | --- |
|  |  | **β-95%CI** | ***P-value*** | **β-95%CI** | ***P-value*** | **β-95%CI** | ***P-value*** | **β-95%CI** | ***P-value*** |
| **Overall** |  |  |  |  |  |  |  |  |  |
| **Dietary folate**  **intake (100ug)** | 6343 | 0 (0~0.01) | 0.036 | 0 (0~0.01) | 0.374 | 0 (0~0.01) | 0.239 | 0 (0~0.01) | 0.74 |
| **Quartile** |  |  |  |  |  |  |  |  |  |
| **Q1** | 1539 | 0(Ref) |  | 0(Ref) |  | 0(Ref) |  | 0(Ref) |  |
| **Q2** | 1607 | 0.01 (-0.01~0.04) | 0.303 | 0 (-0.02~0.03) | 0.761 | 0.01 (-0.02~0.03) | 0.605 | 0.01 (-0.02~0.03) | 0.662 |
| **Q3** | 1592 | 0.01 (-0.02~0.03) | 0.545 | 0.01 (-0.02~0.03) | 0.636 | 0.01 (-0.02~0.03) | 0.456 | 0.01 (-0.02~0.04) | 0.527 |
| **Q4** | 1605 | 0.05 (0.02~0.08) | <0.001 | 0.03 (0~0.06) | 0.02 | 0.03 (0.01~0.06) | 0.009 | 0.03 (0~0.07) | 0.032 |
| ***P* for trend** |  |  | 0.001 |  | 0.029 |  | 0.012 |  | 0.048 |
| **Dietary folate intake** |  |  |  |  |  |  |  |  |  |
| **＜400ug** | 3905 | 0(Ref) |  | 0(Ref) |  | 0(Ref) |  | 0(Ref) |  |
| **≥400ug** | 2438 | 0.04 (0.02~0.06) | <0.001 | 0.03 (0.01~0.05) | 0.003 | 0.03 (0.01~0.05) | 0.001 | 0.03 (0.01~0.05) | 0.004 |

Note:Model 1was not adjusted. Model 2 was adjusted for sociodemographic variables (age and gender). Model 3 was adjusted for Model 2+race, education level, marital status, family PIR, Model 4 was adjusted for Model 3+smoking status, physical activity,BMI and energy consumption.

Abbreviations: LTL, leukocyte telomere length; CI, confidence interval;Q,quartile of dietary folate intake.

**Supplementary Table S2 Subgroup Analyses of the Association between Dietary Folate (≥400 µg/day vs. <400 µg/day) and LTL.**

| **Subgroup** | **Dietary Folate** | **No.of**  **total** | **Crude β-95%CI** | **Crude P-value** | **Adjusted β-95%CI** | **Adjusted P-value** | **P for interaction** |
| --- | --- | --- | --- | --- | --- | --- | --- |
| **Gender** |  |  |  |  |  |  | 0.481 |
| **Male** |  |  |  |  |  |  |  |
|  | <400 µg/day | 1883 | 0(Ref) |  | 0(Ref) |  |  |
|  | ≥400 µg/day | 1637 | 0.05 (0.02~0.07) | <0.001 | 0.07 (0.04~0.09) | <0.001 |  |
| **Female** |  |  |  |  |  |  |  |
|  | <400 µg/day | 2636 | 0(Ref) |  | 0(Ref) |  |  |
|  | ≥400 µg/day | 1168 | 0.05 (0.02~0.07) | 0.001 | 0.02 (-0.01~0.05) | 0.132 |  |
| **Age(years)** |  |  |  |  |  |  | 0.648 |
| **<50 years** |  |  |  |  |  |  |  |
|  | <400 µg/day | 2299 | 0(Ref) |  | 0(Ref) |  |  |
|  | ≥400 µg/day | 1621 | 0.03 (0.01~0.06) | 0.006 | 0.04 (0.02~0.07) | 0.002 |  |
| **≥50 years** |  |  |  |  |  |  |  |
|  | <400 µg/day | 2220 | 0(Ref) |  | 0(Ref) |  |  |
|  | ≥400 µg/day | 1184 | 0.05 (0.03~0.07) | <0.001 | 0.04 (0.02~0.07) | 0.001 |  |
| **Smoking Status** |  |  |  |  |  |  | 0.381 |
| **Never** |  |  |  |  |  |  |  |
|  | <400 µg/day | 2324 | 0(Ref) |  | 0(Ref) |  |  |
|  | ≥400 µg/day | 1454 | 0.06 (0.03~0.08) | <0.001 | 0.04 (0.01~0.07) | 0.004 |  |
| **Former** |  |  |  |  |  |  |  |
|  | <400 µg/day | 1158 | 0(Ref) |  | 0(Ref) |  |  |
|  | ≥400 µg/day | 809 | 0.07 (0.04~0.1) | <0.001 | 0.05 (0.02~0.09) | 0.002 |  |
| **Current** |  |  |  |  |  |  |  |
|  | <400 µg/day | 1037 | 0(Ref) |  | 0(Ref) |  |  |
|  | ≥400 µg/day | 542 | 0.02 (-0.02~0.06) | 0.32 | 0.03 (-0.01~0.07) | 0.141 |  |
| **BMI (kg/m^2^ )** |  |  |  |  |  |  | 0.405 |
| **Normal** |  |  |  |  |  |  |  |
|  | <400 µg/day | 1373 | 0(Ref) |  | 0(Ref) |  |  |
|  | ≥400 µg/day | 929 | 0.05 (0.02~0.09) | 0.001 | 0.06 (0.02~0.09) | 0.001 |  |
| **Overweight** |  |  |  |  |  |  |  |
|  | <400 µg/day | 1586 | 0(Ref) |  | 0(Ref) |  |  |
|  | ≥400 µg/day | 1084 | 0.03 (0~0.06) | 0.051 | 0.03 (0~0.06) | 0.08 |  |
| **Obese** |  |  |  |  |  |  |  |
|  | <400 µg/day | 1560 | 0(Ref) |  | 0(Ref) |  |  |
|  | ≥400 µg/day | 792 | 0.05 (0.02~0.08) | 0.001 | 0.05 (0.01~0.08) | 0.005 |  |

Note: Crude model was not adjusted. Adjusted model was adjusted for sociodemographic variables (age and gender),race, education level, marital status, family PIR,smoking status, physical activity,BMI and energy consumption.

Abbreviations: BMI, body mass index;LTL, leukocyte telomere length ; CI, confidence interval.
